# Association between pre-treatment computed tomography findings and post-treatment persistent decrease in lung perfusion blood volume

**DOI:** 10.1038/s41598-024-62890-7

**Published:** 2024-05-24

**Authors:** Tetsuhiro Hirata, Norihiko Yoshimura, Takuya Yagi, Motohiko Yamazaki, Yosuke Horii, Hiroyuki Ishikawa

**Affiliations:** 1 Department of Radiology and Radiation Oncology, Niigata University Graduate School of Medical and Dental Sciences, 1-757 Asahimachi-Dori, Chuo-Ku, Niigata-City, Niigata 951-8510 Japan; 2 Department of Radiology, Niigata City General Hospital, 463-7 Syumoku, Chuo-Ku, Niigata-City, Niigata 950-1141 Japan

**Keywords:** Cardiology, Cardiovascular diseases

## Abstract

The purpose of this study was to evaluate pre-treatment CT findings in patients with acute pulmonary embolism (PE) and determine the imaging findings associated with residual hypoperfused segments in post-treatment lung perfused blood volume (LPBV). We evaluated 91 patients with acute PE who underwent dual-energy CT before and after treatment. The location of thrombi (proximal or distal) and patency of the pulmonary artery (occlusive or non-occlusive) were recorded using pre-treatment computed tomography pulmonary angiography (CTPA). Residual hypoperfusion was defined as a perfusion-decreased area seen in both the pre- and post-treatment LPBVs. The association of the location of the thrombus and vascular patency of pre-treatment CTPA with residual hypoperfusion on a segmental and patient basis was examined. In the segment-based analysis, the proportion of residual hypoperfusion in the proximal group was significantly higher than that in the peripheral group (33/125 [26.4%] vs. 9/87 [10.3%], P = 0.004). Patient-based analysis also showed that the proportion of residual hypoperfusion in patients with pre-treatment proximal thrombus was significantly higher than those without (16/42 [38.1%] vs. 3/25 (12.0%); P = 0.022). Pre-treatment vascular patency was not significantly associated with residual hypoperfusion (P > 0.05). Therefore, careful follow-up is necessary, especially in patients with proximal thrombi.

## Introduction

Acute pulmonary embolism (PE) has a high mortality rate of 7.2%^1^. Patients with acute PE develop varying degrees of permanent cardiopulmonary dysfunction and other sequelae after treatment. Moreover, some may progress to chronic thromboembolic pulmonary hypertension (CTEPH). CTEPH is caused by organised blood clots and remodelling, and its incidence is reported to be 0.1–9.1% in patients with symptomatic acute PE^2,3^. Moreover, it can lead to heart failure and a poor prognosis if proper treatment is not provided. Although recurrent PE/deep venous thrombosis (DVT) and malignancies have been reported to be risk factors for CTEPH, early diagnosis remains difficult^4,5^.

Computed tomography pulmonary angiography (CTPA) and lung perfusion scintigraphy are conventionally used to assess severity and follow-up post-treatment in cases of acute PE. Dual-energy computed tomography (DECT) has also been used in recent years. DECT uses two different tube voltages and can image the distribution of iodine contrast medium which reflects the blood perfusion of the lungs (lung perfusion blood volume [LPBV]). LPBV is based on three-material decomposition using iodine, air, and soft tissue^6^. Previous studies have shown a good agreement between LPBV and lung perfusion scintigraphy^7,8^. There are also studies on the application of DECT in assessing the severity and predicting the prognosis of acute PE^9,10^. DECT can evaluate morphological information, such as thrombus and perfusion information, in a single examination. DECT provides useful additional information, although the exposure of the patient to additional radiation is not much higher than that of a conventional CTA^11^.

After systemic anticoagulation in cases of acute PE, residual thrombus and areas of hypoperfusion have been reported on imaging examinations, such as pulmonary blood flow scintigraphy and DECT^12,13^. A segmental defect on perfusion scintigraphy due to abnormalities in pulmonary blood flow distribution and pulmonary circulation is observed in patients with CTEPH. Determining the pre-treatment image features associated with residual hypoperfusion areas may provide clues for detecting CTEPH and other sequelae. However, these imaging findings have not yet been clarified. In addition, few studies have evaluated the changes in LPBV before and after treatment^14,15^.

Thus, this study aimed to evaluate pre-treatment CTPA and LPBV findings in patients with acute PE and determine the imaging findings associated with residual decreased segments in post-treatment LPBV.

## Materials and methods

### Ethics

The institutional review board of Niigata University approved this study and waived the requirement for informed consent because of the observational and retrospective nature of the study. All methods were performed in according with the principles of the Declaration of Helsinki.

### Patients

A total of 124 patients with acute PE who underwent CTPA using DECT before treatment and follow-up CTPA using DECT after treatment between September 2009 and March 2018 at our institution were enrolled. However, we excluded 33 patients because of extravasation of the contrast medium into the subcutaneous spaces (n = 2), insufficient injection rates of contrast medium (n = 1), massive pleural effusion or atelectasis (n = 19), obvious increase in thrombus after treatment (n = 7), and strong artefacts (n = 4). Finally, 91 patients (34 men and 57 women; mean age ± standard deviation = 63.7 ± 12.7 years, range 19–87 years; interval between the examinations before and after treatment was 5 ~ 1655 days, mean 122 days, median 55 days) were included in this study. The patients received the following treatments: heparin (65.9% [60/91]), warfarin (65.9% [60/91]), direct oral anticoagulants (31.9% [29/91]), thrombolytic agent (3.3% [3/91]), and implanted IVC filters (20.9% [19/91]).

### Computed tomography (CT) imaging protocol

Two dual-source CT scanners (SOMATOM Definition Flash or SOMATOM Force; Siemens, Germany) were used to examine all patients. The non-ionic contrast media used were iopamidol (370 mgI/mL; Bayer Healthcare, Germany) or iomeprol (350 mgI/mL; Eisai, Japan). An automated power injector was used to administer 100 mL of non-ionic contrast medium at a flow rate of 22.2 mgI/kg/s and an additional 40-mL bolus of 0.9% saline solution at the same flow rate through a 22-gauge catheter.

The automatic bolus tracking method with a region of interest (ROI) placed on the pulmonary artery trunk, which triggered 250 Hounsfield units (HU), was used, and the CTPA scan of CTPA started 10 s after the trigger.

The following parameters were used in the SOMATOM Definition Flash: tube voltage, 140 and 100 kV; detector collimation, 2 × 64 mm × 0.6 mm; rotation time, 330 ms; and pitch, 0.55. The following parameters were used in the SOMATOM Force: tube voltage, 150 and 90 kV; detector collimation, 2 × 64 mm × 0.6 mm; rotation time, 280 ms; and pitch, 0.55. Lung iodine distribution (iodine map) and LPBV images were generated using a workstation (Syngo Acquisition Workplace; Siemens, Germany). LPBV images were created by the fusion of iodine maps and lung CTPA images.

### Analysis of CTPA images

Images were evaluated using a picture archiving and communication system (Synapse, Fujifilm, Japan). Image evaluation was performed twice using images obtained before treatment and at the most recent examination after treatment. The location of the thrombi from the bilateral main pulmonary arteries to the subsegmental pulmonary arteries during pre-treatment CTPA was recorded. The patency of the pulmonary artery was also evaluated, and thrombi were classified into occlusive or non-occlusive. A non-occlusive thrombus was defined as the presence of contrast lumen around the thrombus. An occlusive thrombus was defined as the absence of contrast lumen around the thrombus. We also recorded whether the thrombi detected on pre-treatment CTPA remained in post-treatment CTPA. Image evaluations were performed independently by two radiologists (#1: 10 years of cardiovascular imaging experience; #2: 5 years of cardiovascular imaging experience). If there were disagreements between the radiologists, a final decision was reached by consensus.

### Analysis of LPBV images

**Figure 1:**
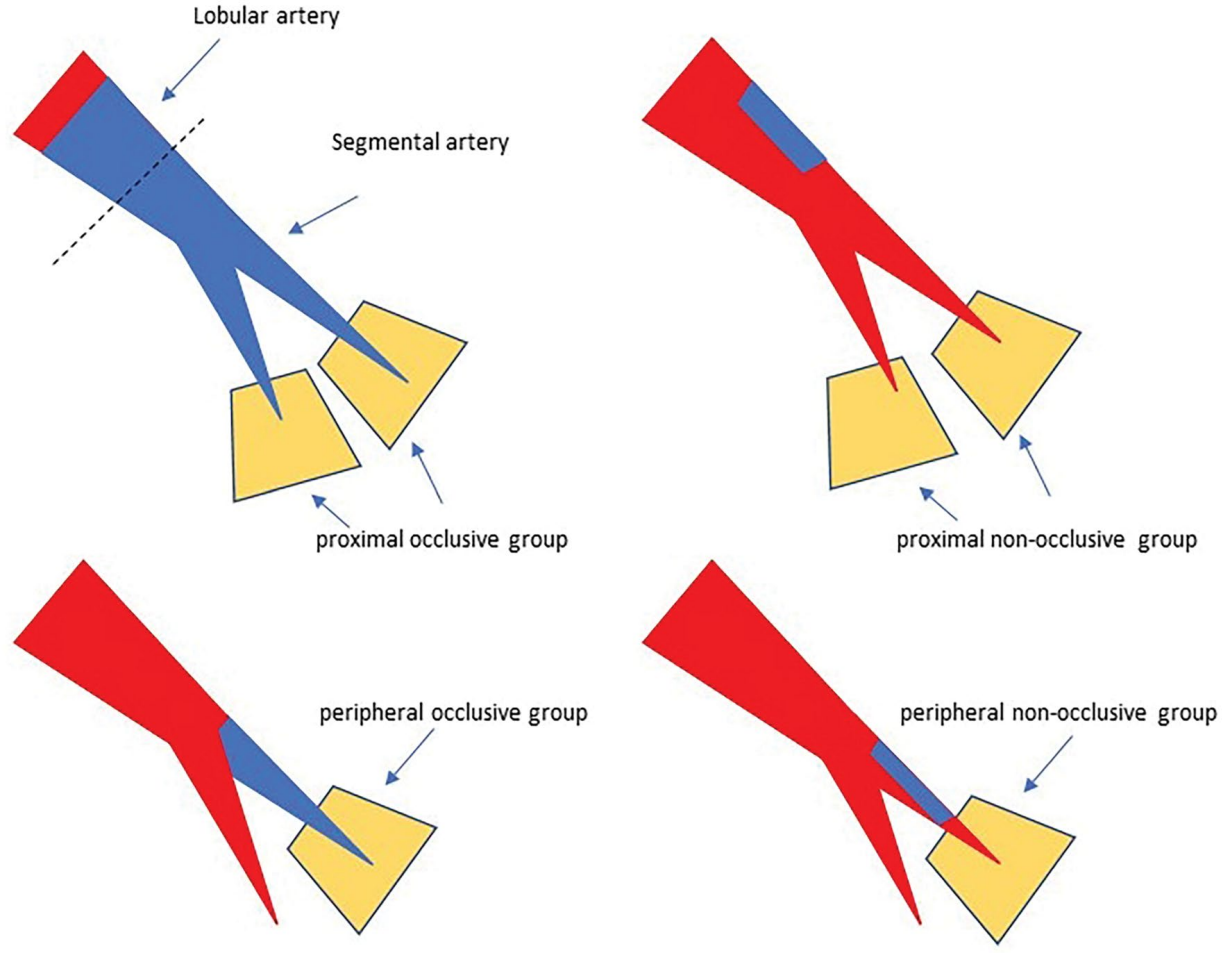
Definition of the segments to be evaluated. The red area is the blood flow cavity, and the blue area is the thrombus. Each evaluated segment was classified into a proximal or peripheral group according to the location of the thrombus and into an occlusive or non-occlusive group according to vascular patency. When thrombi had both proximal and peripheral sides of the segmental artery, the segments were classified into the proximal group.

**Figure 2:**
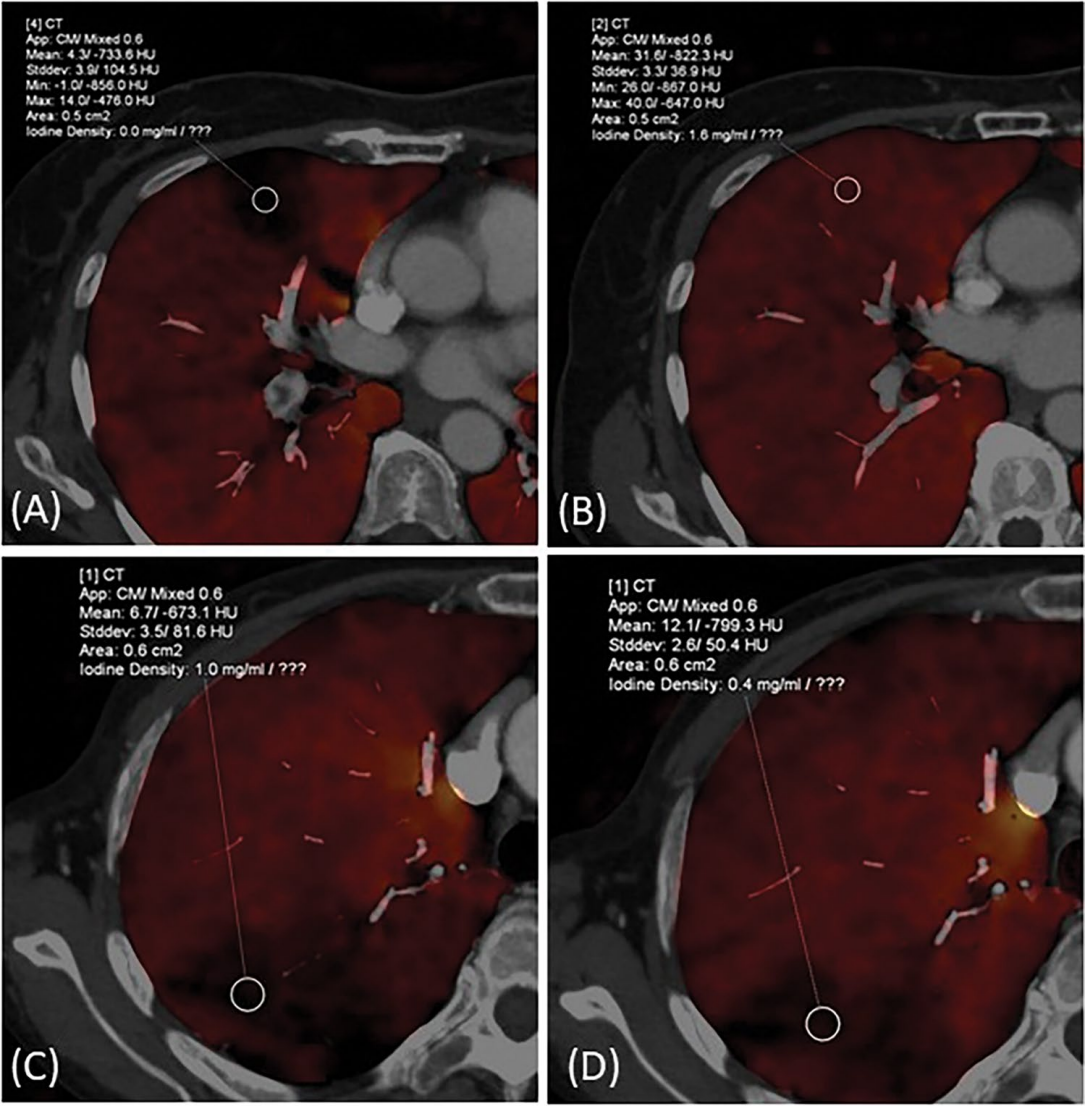
Examples of improved segment; (**A**,**B**) (a 61-year-old woman) and residual hypoperfused segment; (**C**,**D**) (a 51-year-old man). (**A**) Lung perfusion blood volume (LPBV) before treatment shows a perfusion decrease in the right S3. (**B**) The perfusion improved after treatment. (**C**) LPBV before treatment shows a perfusion decrease in the right S2. (D) Perfusion decrease remains even after treatment.

**Figure 3:**
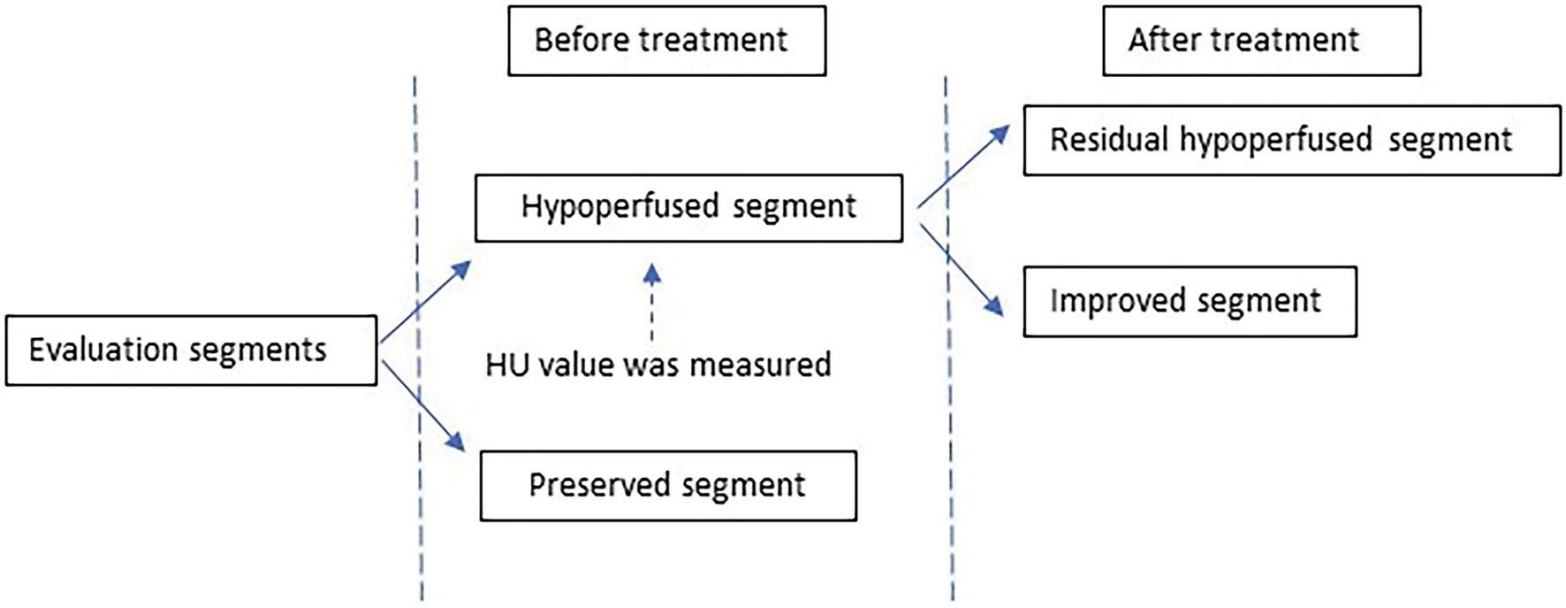
Process of lung perfusion blood volume evaluation.

One radiologist (same as #2 above) evaluated two LPBV images before treatment and at the most recent examination after treatment using a workstation software (Syngo via, Siemens Healthcare). LPBV images were evaluated for each downstream segment (or segment) of the thrombus. Figure 1 shows the classification of each segment. According to the location of the thrombus, the segment downstream of the proximal thrombus was classified as the proximal group, and the segment downstream of the peripheral thrombus was classified as the peripheral group. Cases where segments had both proximal and peripheral thrombi were defined as proximal groups. Therefore, the number of evaluated segments differed from the number of thrombi recorded. Furthermore, according to vascular patency (occlusive or non-occlusive), the downstream segments were classified into the occlusive and non-occlusive groups. For example, if a thrombus completely occluded the middle lobe branch of the right pulmonary artery, the downstream segments to be evaluated are S4 and S5, and both segments are classified into proximal and occlusive groups. The segments were also classified into hypoperfused and preserved segments using pre-treatment LPBV. Furthermore, the hypoperfused segments were classified into improved and residual hypoperfused segments using post-treatment images. A residual hypoperfused segment was defined as a segment with decreased perfusion in both the pre- and post-treatment images. Examples of segment classification are shown in Fig. 2. Furthermore, pre-treatment HU values were quantitatively evaluated by placing a ROI of 50–100 mm^2^ at the centre of the evaluated segments, not overlapping any blood vessels. The LPBV evaluation process is illustrated in Fig. 3.

### Data analysis

#### Segment-based analysis

CT findings (the location of the thrombi, the patency of the pulmonary artery, and HU values of LPBV of the pre-treatment image) and clinical information (age, gender, the interval between the examinations before and after treatment, treatment given to patients) that were significantly associated with the residual hypoperfused segments in post-treatment LPBV were determined. Categorical variables were statistically analysed with the χ-square test and continuous variables with the t-test. Factors with a p-value < 0.10 in univariate analysis were evaluated by multivariate logistic regression analysis to determine the independent factor associated with residual decreased segments in post-treatment LPBV.

Chi-square test was also performed to compare the proportion of residual thrombi in the residual decreased groups between the occlusive and non-occlusive groups and between the proximal and peripheral groups.

#### Patient-based analysis

A χ-square test was used to determine whether the percentage of patients with a residual hypoperfused segment was significantly associated with the percentage of patients with a segment of a proximal or an occlusive group.

#### The software used for analysis

Statistical significance was set at p < 0.05. IBM SPSS Statistics for Windows, version 25 (IBM Corp., Armonk, N.Y., USA) was used for analysis.

## Results

### Segment-based analysis

On pre-treatment CTPA images, a total of 890 thrombi were detected, and 738 downstream segments of these thrombi were included in the evaluation.

The association between pre-treatment CTPA and LPBV is shown in Table 1. Among the 738 segments, 212 were hypoperfused, and 526 were preserved. Among the 212 hypoperfused segments, 87 (41.0%) were in the peripheral group, and 125 (59.0%) were in the proximal group. According to vascular patency, 127 (59.9%) hypoperfused segments were classified into the occlusive group, and 85 (40.1%) hypoperfused segments were classified into the non-occlusive group.

**Table 1:** Association between CTPA and LPBV findings before treatment (segment-based analysis).

Pretreatment CTPA findings (n = 738)	Pretreatment LPBV findings	
	Hypoperfused segment (n = 212)	Preserved segment (n = 526)
Location of thrombus		
Peripheral group (n = 237)	87 (41.0%)	150 (28.5%)
Proximal group (n = 501)	125 (59.0%)	376 (71.5%)
Vascular patency		
Occlusive group (n = 183)	127 (59.9%)	56 (10.6%)
Non-occlusive group (n = 555)	85 (40.1%)	470 (89.4%)

CTPA, computed tomography pulmonary angiography; LPBV, lung perfusion blood volume.

**Figure 4:**
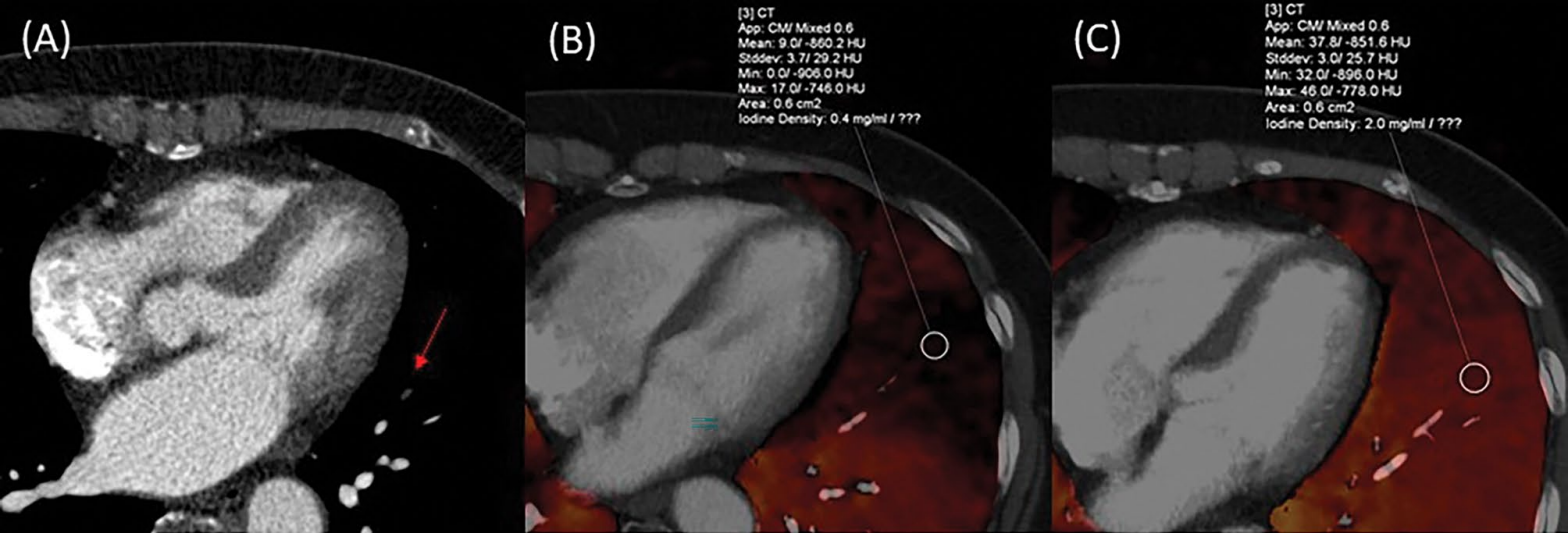
A 51-year-old-man with pulmonary embolism. (**A**) Peripheral occlusive thrombi can be seen in left A4a on the pre-treatment computed tomography pulmonary angiography (CTPA). (**B**) Pre-treatment perfused blood volume shows perfusion decrease in the left S4a. The mean HU value of the segment is 9.0 HUs. (**C**) Lung perfusion blood volume improved after treatment. The mean computed tomography value of the segment is 37.8 HUs.

**Figure 5:**
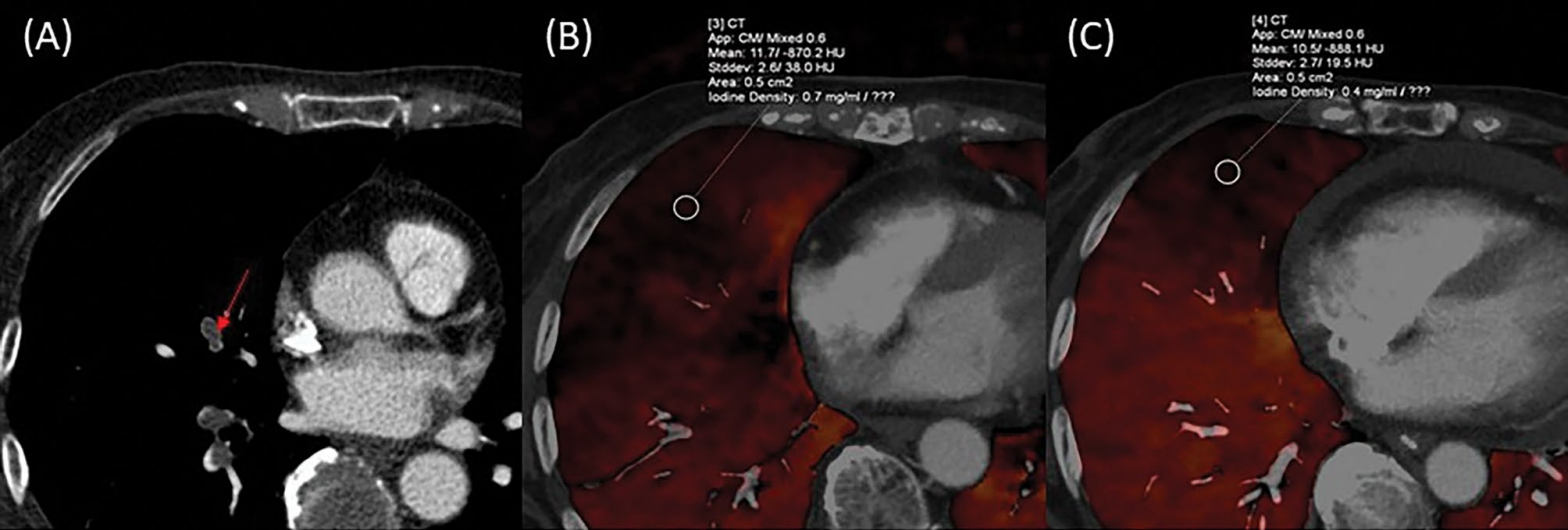
A 68-year-old man with pulmonary embolism. (**A**) Pre-treatment computed tomography pulmonary angiography indicates central occlusive thrombus from the right middle lobar pulmonary artery to A5. (**B**) Pre-treatment perfused blood volume shows perfusion decrease in right S5. The mean CT value of the iodine map is 11.7 HUs. (**C**) Lung perfusion blood volume remains decreased even after treatment. Mean CT value of the segment is 10.5 HUs.

Univariate analysis to determine the predictors of residual hypoperfused segments is shown in Table 2. Among the 212 pre-treatment hypoperfused segments, 42 (19.8%) segments were classified under residual hypo-perfused segment after treatment. The proportion of residual hypoperfused segments was significantly higher in the proximal group than in the peripheral group (26.4% [33/125] vs. 10.3% [9/87], P = 0.004) (Figs. 4 and 5). Vascular patency before treatment was not significantly related to the proportion of residual hyperperfused segments (P = 0.56). Pre-treatment HU values measured on LPBV images were not significantly different between the improved and residual hypoperfused segments (9.156 ± 6.3262 HUs and 9.469 ± 7.567 HUs, P = 0.783). The age and sex ratios were not significantly different (p > 0.05 for both). The interval of CT examinations before and after treatment were significantly different between the improved and residual hypoperfused segments (55.0 ± 78.5 days and 95.1 ± 127.6 days, P = 0.01). Concerning the treatments used, significant differences were observed for thrombolytic therapy (P = 0.012), but none of the other treatments (heparin, warfarin, DOAC, and inferior vena cava filters) were significantly different between the improved and residual hypoperfused segments (p > 0.05 for all).

**Table 2:** Univariate analysis to determine the predictors for residual hypoperfused segments.

Variables	Univariate analysis		
	Improved segment (n = 170)	Residual hypoperfused segment (n = 42)	P-value
Location of the thrombi^a^			0.004
Peripheral thrombus	78/87 (89.7%)	9/87 (10.3%)	
Proximal thrombus	92/125 (73.6%)	33/125 (26.4%)	
Vascular patency^a^			0.563
Occlusive thrombus	101/127 (79.5%)	26/127 (20.4%)	
Non-occlusive thrombus	69/85 (81.2%)	16/85 (18.8%)	
HU values of LPBV^b^	9.156 ± 6.3262	9.469 ± 7.567	0.783
Age^b^	64.1 ± 13.5	63.9 ± 12.0	0.942
Sex ratio (male: female)^a^	65: 105	16: 26	0.987
Follow-up interval (days)^a^	95.05 ± 127.6	55.02 ± 78.49	0.011
Heparin therapy (n = 142)^a^	113/142 (79.6%)	29/142 (20.4%)	0.75
Warfarin therapy (n = 126)^a^	105/126 (83.3%)	21/126 (16.7%)	0.164
DOAC (n = 83)^a^	64/83 (77.1%)	19/83 (22.9%)	0.367
Thrombolytic therapy (n = 7)^a^	3/7 (42.9%)	4/7 (57.1%)	0.012
IVC filter detention (n = 47)^a^	33/47 (70.2%)	14/47 (29.8%)	0.052

DOAC, direct oral anticoagulant; IVC, inferior vena cava; HU, Hounsfield units; LPBV, lung perfusion blood volume.

^a^χ-square test.

^b^t-test.

The results of the Multivariate logistic regression analysis are presented in Table 3. Multivariate logistic regression analysis showed that proximal thrombus (odds ratio, 2.617; 95% CI 1.152–5.945; p = 0.022) and the thrombolytic agent (odds ratio 5.237, 0; 95% CI 1.066–25.737; p = 0.042) was significantly associated with residual hypoperfused segments.

**Table 3:** Multivariate logistic regression analysis to determine the independent predictor of residual hypoperfused segments.

Variables	Multivariate analysis	
	OR (95% CI)	P-value
Location of the thrombi^a^		
Proximal thrombus	2.617 (1.152‒5.945)	0.022
Follow-up interval (days)^a^	0.997 (0.993‒1.001)	0.115
Thrombolytic therapy(n = 7)^a^	5.237 (1.066‒25.737)	0.042
IVC filter detention (n = 47)^a^	1.639 (0.745‒3.604)	0.219

Only variables with P < 0.10 in univariate analysis were included in multivariate analysis. CI, confidence interval; OR, odds ratio.

**Figure 6:**
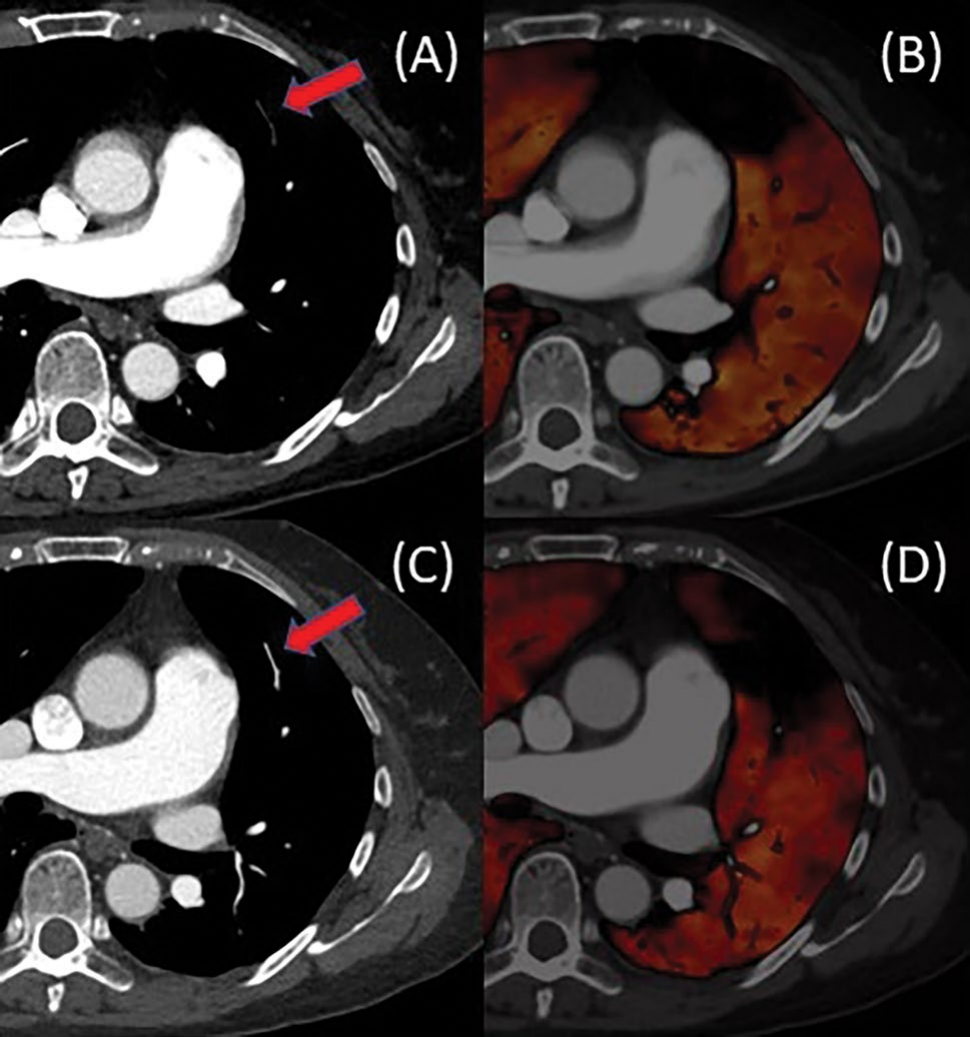
A 66-year-old woman with pulmonary embolism. (**A**) Pre-treatment computed tomography pulmonary angiography (CTPA) indicates peripheral occlusive thrombi of Lt. A4b. (**B**) Pre-treatment perfused blood volume shows perfusion decrease. (**C**) The thrombi disappeared on post-treatment CTPA. (**D**) Although the thrombi disappeared, the perfusion decrease remains on post-treatment lung perfusion blood volume.

Table 4 shows the association between pre- and post-treatment CTPA findings in the residual hypoperfused segment (n = 42). The proportion of residual thrombi after treatment was significantly higher in the proximal group than in the peripheral group (78.8% [26/33] vs. 44.4% [4/9] P < 0.001). However, no significant association was found between vascular patency before treatment and residual thrombi after treatment (P = 0.791). Furthermore, 12 of the 42 residual hypoperfused segments did not have thrombi (Fig. 6).

**Table 4:** Association between pre-and posttreatment CTPA findings of residual decreased groups (segment -based analysis).

Pretreatment CTPA findings* (n = 42)	Posttreatment CTPA findings		P-value
	Segments with thrombus disappeared (n = 12)	Segments with residual thrombus (n = 30)	
Location of thrombus			< 0.001
Peripheral group (n = 9)	5 (55.6%)	4 (44.4%)	
Proximal group (n = 33)	7 (21.2%)	26 (78.8%)	
Vascular patency			0.791
Occlusive group (n = 26)	7 (26.9%)	19 (73.1%)	
Non-occlusive group (n = 16)	5 (31.3%)	11 (68.8%)	

*Findings of residual hypoperfused segments.

CTPA, computed tomography pulmonary angiography.

### Patient-based analysis

The results of the patient-based analysis are presented in Table 5. Sixty-seven out of the 91 patients had at least one hypoperfused segment before treatment. Among the 67 patients, 19 had at least one residual hypoperfused segment, even after treatment. Patients with proximal thrombi before treatment had residual hypo-perfused segments more frequently than those without (16/42 [38.1%] vs. 3/25 [12.0%]; P = 0.022). As with segment-based analysis, pre-treatment vascular patency was not significantly associated with residual hypoperfused segments after treatment (P = 0.148).

**Table 5:** Association between pretreatment CTPA and posttreatment LPBV findings (patient-based analysis).

Pretreatment CTPA findings * (n = 67)	Posttreatment LPBV findings		P-value
	Patients without residual hypoperfused segments (n = 48)	Patients with residual hypoperfused segments (n = 19)	
Location of thrombus			0.022
Patient without proximal thrombi (n = 25)	22 (88.0%)	3 (12.0%)	
Patient with proximal thrombi (n = 42)	26 (61.9%)	16 (38.1%)	
Vascular patency			0.148
Patient without occlusive thrombi (n = 8)	4 (50.0%)	4 (50.0%)	
Patient with occlusive thrombi (n = 59)	44 (74.6%)	15 (25.4%)	

*Findings of patients with hypoperfused segments.

IVC, inferior vena cava.

## Discussion

This study demonstrated that the presence of residual hypoperfused segments in post-treatment LPBV were more likely to be observed in cases where the proximal thrombus was present before treatment, regardless of the interval between the examinations before and after treatment. The results of patient-based analysis also showed that residual hypoperfused segments in post-treatment LPBV were more likely to be observed in patients with proximal thrombi.

Patients treated with a thrombolytic agent were more likely to have residual hypoperfusion segments. This result may have been influenced by the severity of the acute PE. Namely, patients treated with the thrombolytic agent probably had more severe PE than those without the thrombolytic agent.

After anticoagulation therapy in acute PE, residual areas of hypoperfusion have been reported on pulmonary blood flow scintigraphy^12^. The clinical significance of residual areas of hypoperfusion remains unclear, but it has been reported to correlate with increased pulmonary artery pressure and functional limitations, such as dyspnea, and to be a predictor of recurrent venous thromboembolism^16,17^. As LPBV findings correlate well with pulmonary perfusion scintigraphy findings^7,8^, the residual hypoperfusion areas observed in this study may also be related to these sequelae and recurrent venous thromboembolism. Further investigation is needed to determine if the residual hypoperfusion areas in LPBV are related to patient prognosis.

We also found that the proportion of post-treatment residual thrombi in the residual hypoperfused segment was significantly higher in the proximal group than in the peripheral group. Therefore, the residual thrombus may account for the presence of residual hypoperfused segments in post-treatment LPBV in the proximal group. We assume that since proximal vessels have a higher thrombus volume than peripheral vessels, the thrombi in the proximal vessels may be more likely to remain even after treatment. Consistent with these results, previous studies have shown that proximal thrombi are more likely to remain than peripheral thrombi^18,19^.

In this study, thrombi were not visually detected in 12 out of the 42 residual hypoperfused segments. Similar cases have been previously reported by Sven et al.^7^ Researchers assumed that undetected microthrombi on CTPA could be a possible cause of hypoperfusion in LPBV. The residual hypoperfused segments where no thrombus was identified after treatment may have been caused by migration of the undissolved microthrombus into the peripheral pulmonary artery. Another possible cause is that residual fibrous tissue or vessel stenosis after thrombolysis was not detected in CTPA. CTPA has a limited ability for detecting residual hypoperfused regions. Although pulmonary artery perfusion scintigraphy can detect these residual hypoperfusion lesions, it requires more scanning time than a CT; thus, it cannot be a readily available tool for the follow-up of patients with acute PE. In contrast, DECT can be performed in a short time, and LPBV agrees well with pulmonary blood flow by scintigraphy, which is the gold standard for pulmonary blood flow evaluation^7,8^. In addition, DECT can provide more information than pulmonary perfusion scintigraphy because it can evaluate morphological information, such as the location and nature of the thrombi and presence of DVT and malignancy in a single examination. Therefore, the addition of LPBV to CTPA may provide additional information for PE follow-up compared with CTPA alone.

However, LPBV should be carefully evaluated because it may be affected by various factors. One of the most important factors influencing LPBV is the imaging protocol. The contrast medium, dosage, and injection rate vary among institutions, and there is no fixed protocol. Another possible factor that can affect LPBV is the changes in pulmonary circulation^20^. For example, collateral blood flow from the systemic circulatory system may improve LPBV. The development of collateral blood flow from the systemic circulation, such as bronchial arteries, is more likely to occur in patients with chronic pulmonary embolism than in those with acute PE^21,22^. Collateral flow may compensate for the blood flow in the pulmonary artery and improve hypoperfused segments in the LPBV. Some improved segments after treatment in our study may have been affected by the collateral blood flow. Cardiac dysfunction has also been reported to be a long-term complication of acute PE^23^. This may also affect the distribution of contrast medium in the pulmonary vessels.

Nevertheless, our study had several limitations. First, this was a single-centre, retrospective study. Second, the interval of CT examinations before and after treatment varied. Third, we did not examine the severity of acute PE such as right ventricular overload. Fourth, we did not assess the change in volume of the hypoperfusion area before and after treatment. Fifth, the number of cases was relatively small. Sixth, we did not examine whether any patient developed CTEPH because long-term observations were not performed.

In conclusion, residual hypoperfused segments in post-treatment LPBV were associated with the presence of pre-treatment proximal thrombus. Nevertheless, whether the presence of proximal thrombus may help predict the development of CTEPH and other sequelae should be further investigated.

## Date availability

The data used in this study are available from the corresponding author upon reasonable request.
